# Antiulcerogenic effect of *Cuphea ignea* extract against ethanol-induced gastric ulcer in rats

**DOI:** 10.1186/s12906-019-2760-9

**Published:** 2019-12-02

**Authors:** Amria M. Mousa, Nermin M. El-Sammad, Sherien K. Hassan, Abd El Nasser A. Madboli, Amani N. Hashim, Eman S. Moustafa, Sherien M. Bakry, Elsayed A. Elsayed

**Affiliations:** 1 Department of Biochemistry, National Research Centre, Dokki, Cairo, Egypt; 2 Department of Animal Reproduction and Artificial Insemination Research, National Research Centre, Dokki, Cairo, Egypt; 3 Department of Phytochemistry and Plant Systematics, National Research Centre, Dokki, Cairo, Egypt; 4 October University of Modern Sciences and Arts, 6th October City, Egypt; 5 Bioproducts Research Chair, Zoology Department, College of Science, King Saud University, Riyadh, Kingdom of Saudi Arabia; 6 Department of Chemistry of Natural and Microbial Products, National Research Centre, Dokki, Cairo, Egypt

**Keywords:** Gastric ulcer, Antioxidants, Oxidative stress, *Cuphea ignea*, Histopathology, Phenolic compounds

## Abstract

**Background:**

*Cuphea ignea* is one of the herbal resources belonging to Lythraceae family. Some species of this family have been used traditionally in South and Central America’s folk medicine for treating stomach disorders. Therefore, the present study was performed to evaluate the gastropreventive effect of aqueous ethanolic extract of *C. ignea* aerial parts on ethanol-induced gastric ulcer.

**Methods:**

Gastric ulcers were induced in Sprague Dawley rats using one oral dose of absolute ethanol (1.5 mL/rat). The *C. ignea* aerial parts extract at doses of 250 and 500 mg/kg body weight and ranitidine (a reference drug) at a dose of 30 mg/kg body weight were orally administrated daily for 7 days before ulcer induction. One hour after ethanol administration blood samples were collected and then stomachs of sacrificed rats were subjected to biochemical, macroscopic and microscopic studies.

**Results:**

Oral administration of *C. ignea* extract significantly attenuated gastric ulcer as revealed by significant reduction in the gastric ulcer index and volume of gastric juice while significantly increased preventive percentage, gastric pH value and pepsin activity. Pre-treatment of *C. ignea* extract markedly improved the serum level of TNF-α, the gastric MPO activity and NO content. Furthermore, *C. ignea* pre-treatment significantly increased the gastric levels of enzymatic and non- enzymatic antioxidants namely CAT, SOD, GSH-Px, and GSH with concomitant reduction in MDA level compared with those in the ethanol group. These results were further supported by histopathological findings which revealed the curing effect of *C. ignea* on the hemorrhagic shock induced by ethanol toxicity.

**Conclusions:**

*C. ignea* extract showed a potential gastroprotective effect on ethanol-induced gastric ulcer, and its effect may be mediated through suppression of oxidative stress and gastric inflammation.

## Background

Gastric ulcer is a benign lesion with multiple etiologies, associated with an imbalance between gastric protective factors and aggressive physical, chemical or psychological factors on the mucosal epithelium [1]. These aggressive factors include physical stress, prominent tobacco consumption, alcohol or caffeine, certain types of medications, particularly the non-steroidal anti-inflammatory drugs and infection by *Helicobactor pylori* [2]. Among these factors, high alcohol consumption is the greatest cause of gastric mucosal damage [3]. Thus, the experimental model of ethanol-induced gastric ulcer often employed to screen the anti-ulcer compounds [4].

In spite of the domination of synthetic drugs in managing most of human diseases including gastric ulcer, extensive proportion worldwide now directed to traditional medicine [5]. This may be, in part, due to considerable incidence of side effects, drug interactions, microbial resistance and high cost during chemical therapy [6]. Hence, natural products with wide biological activities, better effectiveness and safe profiles are needed to substitute chemical medications [7, 8]. Consequently, there is extensive require for scientific analysis of herbal products with pharmacological effects to discover alternative bioactive phytocompounds [9].

Plants of Lythraceae family are regarded as a valuable source of exclusive natural products for developing medications against various diseases [10]. *Cuphea*, a new world genus, is considered the largest genera of Lythraceae family [11]. Plants of this genus had been used in the Brazilian folk medicine as an oral contraceptive, hypotensive, diuretic, anti-inflammatory, antipyretic and laxative [12]. Some species of this genus have been used for treating stomach disorders, gonorrhea, syphilis and cancer [13, 14].

*Cuphea ignea*, cigar plant, is a flowering species in genus *Cuphea*. It is a tropical, densely branched evergreen subshrub produces tubular, bright red to orange flowers resemble a lit cigar, hence its name. *C. ignea is* native to Mexico and the West Indies; however, in recent years its popularity is on rise everywhere [15].

So far there are no studies regarding the phytochemistry of *C. ignea* except Bate-Smith [16] who studied the flavonoids of some Lythraceae plants and reported the presence of quercetin and kaempferol glycosides in *C. ignea* plant. Recently, we isolated from this extract a coumarin with a rare structure, namely, 7-hydroxy 3-methoxy coumarin 5-O-β-glucopyranoside, which offered potent antioxidant activities in vitro [17]. To date, there is no report proving the biological activity of *C. ignea* in vivo. Therefore, the present study was undertaken to evaluate phytochemical constituents of the aqueous ethanolic extract of *C. ignea* aerial parts and to estimate its gastroprotective effect of *C. ignea* extract against ethanol induced gastric ulcer in rats.

## Methods

### Plant collection and extract preparation

Fresh samples of *C. ignea* aerial parts were collected from 30 k north Cairo. Authentication of the plant was carried out by Prof. Dr. Salwa Kawashty at the NRC. A voucher specimen was deposited at the herbarium of the NRC (voucher number C 182).The collected *C. ignea* plant was dried in the shadow, crushed and exhaustively extracted with 70% (v/v) aqueous EtOH under reflux. The obtained eluent was dried under vacuum at 55–60 °C then dissolved in EtOH. Then, the extract was stored for future use.

### Phytochemical screening

Phytochemical analysis of the aqueous ethanolic extract was carried out as described by Sofowora [18].

### Estimation of total phenolic content

Folin-Ciocalteu method [19] was used to determine total phenolic contents. Briefly, 100 μL of the extract was transferred into a test tube and the volume adjusted to 3.5 mL with distilled water and oxidized with the addition of 250 μL of Folin-Ciocalteau reagent. After 5 min, the mixture was neutralized with 1.25 mL of 20% aqueous sodium carbonate solution. After 40 min, the absorbance was recorded at 725 nm against blank. A previously prepared gallic acid-calibration curve was used to deduce the contents of total phenolics using the equation: y = 0.024x + 0.018 (R^2^ = 0.998), which represented results as gallic acid equivalents.

### Estimation of total flavonoids

Total flavonoid content in the aqueous ethanolic extract of *C. ignea* plant was determined according to Žilić et al. [20] using aluminum chloride assay. Briefly, 300 μL of 5% sodium nitrite was mixed with 100 μL of extract. After 6 min, 300 μL of a 10% AlCl_3_ solution was added and the volume was adjusted to 2.5 mL using distilled water. After 7 min, 1.5 mL of 1 M NaOH was added, followed by centrifugation (5000 g/10 min). Absorbance of the supernatant was measured at 510 nm against the solvent blank. Total flavonoids were estimated using a catachine calibration curve and the equation: y = 0.003x - 0.004 (R^2^ = 0.998).

### Determination of radical scavenging capacity

The quantitative scavenging of 2,2-diphenyl-1-picrylhydrazyl radical was determined following the Brand et al. method [21]. The extract was dissolved in a concentration of 1 mg/mL in ethanol. From this stock solution, concentrations of regular dilution were prepared. Then 500 mL of sample, 375 mL ethanol and125mL of 1 mmol/L prepared scavenging radical solution were placed together. The test was performed in triplicate. After incubation (30 min/dark/room temperature), absorbance was measured at 517 nm on UV–vis spectrophotometer (Shimadzu, Duisburg, Germany). Ascorbic acid was used as reference standard to conclude the radical scavenging activity percentage (RSA):

$$ RSA\%=\frac{\left( Control\ absorbance- Sample\ absorbance\right)}{\left( Control\ absorbance\right)}x100 $$

### Reducing power assay

The effect of *C. ignea* extract on the reduction of ferric cyanide into ferrous cyanide was evaluated according to Yen and Duh [22]. A serial dilution of the extract was performed (400, 300, 200, 100, 50, 25 and 12.5 μg/mL) in 0.2 M phosphate buffer (pH 6.6) containing 1% ferrocyanate. Tubes containing 5 mL of the mixture were incubated (50 °C/20 min), followed by addition of 2.5 mL of 10% TCA (w/v), and then centrifuged (3000 g/10 min). The absorbance of the separated supernatant (mixed with 2.5 mL distilled water containing/1% FeCl_3_) was measured at 700 nm.

### Acute toxicity study

In order to detect the maximal safe dose, Sprague Dawley rat model was used to investigate the effect of *C. ignea* extract on acute toxicity using OECD 425 guidelines [23]. Thirty rats were randomly divided equally into four groups with each group having 5 rats. Groups 1–3 were orally dosed with varying doses (500; 1000; 3000 and 5000) mg/kg of *C. ignea* extract. Group 6 was given an equivalent volume of distilled water. Animals were evaluated clinically and toxicologically for 3 days after receiving the extract, while death rates were monitored for 14 days.

### Experimental animals and grouping

The experiments were performed on adult female Sprague-Dawley rats (150-200 g) obtained from the animal house colony of the National Research Centre, Dokki, Giza, Egypt. The animals were kept in polypropylene cages with wood shaving under standardized animal house conditions (room temperature: 25 ± 3 °C, 55 ± 5% humidity with 12 h dark/light cycles), fed with standard pellet and allowed free access to water. Distilled water was used for the oral administration of standard drug and plant extracts in all in vivo assays. The animal experiments were conducted according to the international regulations of the usage and welfare of laboratory animals and were approved by the Ethics Committee of the National Research Centre, Cairo, Egypt. Rats were randomized into seven groups (*n* = 6) as follows: Group 1 (normal control rats); Group 2 (ethanol ulcerated rats); Group 3 (ulcerated rats pretreated with reference drug, 30 mg/kg ranitidine); Group 4 (rats administered only 250 mg/kg of extract); Group 5 (ethanol ulcerated rats pretreated with 250 mg/kg of extract); Group 6 (rats were administered only 500 mg/kg of extract); Group 7 (ethanol ulcerated rats pretreated with 500 mg/kg of extract). Ranitidine and extract treatments were given orally once/day for 7 days before ulcer induction. Groups 4 and 6 were used to evaluate the negative/toxicological effects of the extract. Groups 2, 3, 5 and 7 were supplied only with water for 24 h before ulcer induction. A single gavage of absolute ethanol (1.5 mL/rat) was used to induce gastric ulcer after 24 h fasting according to Liu et al. [4].

### Tissue sampling and collection of blood and gastric juice

One hour after the ulcer induction, animals were anesthetized by 1.9% diethyl ether-saturated cotton ball in a small chamber for 2–5 min., and euthanized by cervical dislocation. Blood samples were collected and centrifuged (3000 rpm/10 min), where clear serum was separated and stored at −20 °C until analysis. In parallel, animal stomachs were rapidly taken away, opened along the greater curvature, where their contents were collected for volume and pH determination. The gastric secretion was stored for estimating pepsin activity. Gastric tissue specimens were thereafter rinsed gently with phosphate buffer saline (PBS) to remove any blood clots and then examined macroscopically to calculate gastric ulcer index [6]. Secondly, each stomach was dichotomized, with one moiety of stomach immersed in 10% formaldehyde for histological examination and the other moiety was homogenized in 0.1 M potassium phosphate buffer, pH 7.4 at a ratio of 1:10 (w/v). The homogenates were centrifuged (3000 rpm/10 min/4°C) using 3-18KS Sigma cooling centrifuge, Germany. Myeloperoxidase (MPO) activity was detected in the obtained pellets, while supernatants were stored at − 80 °C for further biochemical investigations.

### Estimation of gastric ulcer index

For each group, ulcer index was determined using the following equation:

$$ Ulcer\ Index=\frac{Sum\  of\ lesion\ area s}{Total\ stomach\ area}\ x100 $$

The percentage of ulcer preventive index was then calculated as follows:

$$ Preventive\ index=\frac{Ulcer\ index\ \left( ulcerated\ control\right)- Ulcer\ index\ (treated)}{Ulcer\ index\ \left( ulcerated\ control\right)}\kern0.37em x\;100 $$

### Estimation of pepsin activity in gastric secretion

Pepsin activity was determined using stop-point assay of denatured hemoglobin hydrolysis [24].

### Biochemical analysis in serum

Serum aspartate transaminase (AST), alanine transaminase (ALT) and alkaline phosphatase (ALP) activities were determined using commercial kits (Spectrum Diagnostics Company, Egypt). Serum urea and creatinine level were assayed as kidney function tests using kits also provided by Spectrum Diagnostics Company, (Egypt). Serum necrosis factor-alpha (TNF-α) was investigated by the enzyme-linked immunosorbent assay using Koma Biotec Inc. kits, Korea. The operational processes were measured in accordance with the kit instructions.

### Biochemical analysis in tissue homogenate

#### Myeloperoxidase activity

Myeloperoxidase, a marker of neutrophil infiltration, was assayed using a modified method of Bradley et al. [25]. In brief, the pellet from gastric homogenate was resuspended in 50 mM potassium phosphate buffer (pH 6.0) containing 0.5% hexadecyl trimethyl ammonium bromide using TM 125 tissue master homogenized (Omni, USA). Three freeze/thaw cycles were then performed followed by 10 s sonication using VCX500 sonicator (Sonics & materials, Inc. USA). Suspensions were centrifuged (4 °C/15 min/15000 rpm), and the supernatant was used to detect MPO activity at 460 nm using o-dianisidinedihydrochloride and 0.005% hydrogen peroxide. One unit of MPO activity was defined as that degrading 1 μmol peroxide/min/25 °C.

#### Nitric oxide assay

Nitric oxide (NO) concentration was assayed by measuring nitrite formed from NO oxidation [26], based on Griess diazotization reaction.

#### Catalase assay

The initial rate of H_2_O_2_ disappearance at 240 nm was used to detect catalase (CAT) activity according to Aebi [27]. Enzyme activity (1 unit) is equivalent to enzyme concentration used to decompose 1 μmol of H_2_O_2_/min/25°C at pH 7.0.

#### Superoxide dismutase assay

Superoxide dismutase (SOD) activity estimated according to method of Minami and Yoshikawa [28], where pyrogallol autoxidation is inhibited through superoxide radical catalysis, reaction with nitro-blue tetrazolium and measurement of formed formazan dye at 540 nm. Enzyme activity (1 unit) is equivalent to enzyme concentration inhibiting 50% of pyrogallol autoxidation.

#### Glutathione peroxidase assay

Glutathione peroxidase (GSH-Px) activity was determined according to method of Necheles et al. [29] at 412 nm. Enzyme activity (1 unit) is equivalent to 1 µmol GSH consumed per minute.

#### Reduced glutathione assay

The method of Beutler et al. [30] was used for GSH assay. Tissue homogenate supernatants was previously treated with equal volumes of 10% (HPO_3_)_n_, then centrifuged for at least 2 min at 4000 rpm to eliminate proteins in order to avoid interferences of protein R-SH groups.

#### Thiobarbituric acid reactive substances assay

Malondialdehyde (MDA), resulting from lipid peroxidation, was assayed [31] based on the reaction of MDA with amino group of thiobarbituric acid forming 1:2 adduct that absorbs strongly at 532 nm.

### Histopathological procedure

Tissue specimens were taken mainly from the glandular part of stomach of the all groups and fixed in 10% neutral buffer formalin overnight. Routine tissue processing was carried out according to Suvarna et al. [32]. Tissue blocks were cut into 3 μm-sections and stained with hematoxylin and eosin (H&E) for histopathological examination.

### Statistical analysis

All results were expressed as means ± SD. The data were evaluated with SPSS 19.0 (SPSS Inc., Chicago, IL, USA). The statistical significance of differences for each parameter among the groups was evaluated by one-way ANOVA, followed by LSD test. The significance level was set at *P* < 0.05.

## Results

### Phytochemical examination

The qualitative phytochemical screening of aqueous ethanolic extract of *C. ignea* plant revealed the presence of phenolics, flavonoids, tannins, alkaloids, carbohydrates, glycosides, triterpenens and unsaturated sterols. The quantitative phytochemical investigation of total phenolic and flavonoid contents in the extract was shown in Table 1.

**Table 1:** Total phenolic (TPC) and total flavonoid (TFC) contents in *C. ignea* aerial parts extract

Total phenols	121.66 mg/g
Total flavonoids	105.33 mg/g

TPC is expressed as milligram of gallic acid equivalent per gram of extract. TFC is expressed as milligrams of catachine equivalents per gram of extract

### Radical scavenging activity of *C. ignea* aerial parts extract

**Fig. 1:**
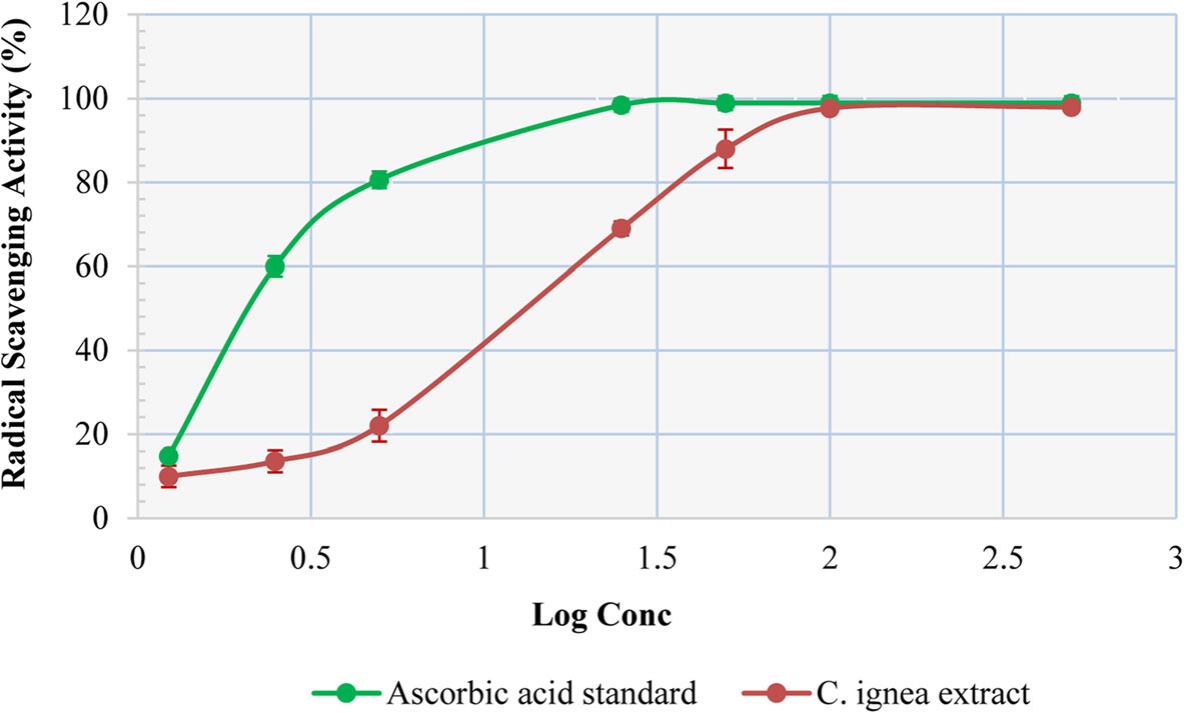
Radical scavenging activity of *C. ignea* aerial parts extract in comparison to ascorbic acid*.* Results are given as mean ± SD of 3 independent experiments. RSA with 98% considered as a full absorbance inhibition of scavenging radical because absorbance inhibition of the final solution will never reach 100%

Radical scavenging assay is used to determine the in vitro antioxidant activity of plant extracts. Figure 1 showed the radical scavenging activity of *C. ignea* extract at seven different concentrations ranging from 0.5 to 500 μg/mL (expressed by log scale) used in comparison with ascorbic acid as standard reference. *C. ignea* extract exhibited nearly similar RSA as ascorbic acid at a concentration of 50 μg/mL, the extract completely inhibited the scavenging radical absorbance and give a potent radical scavenging activity with 98% at concentration of 100 μg/mL, indicating a remarkable antioxidant capacity.

### Reducing capacity of *C. ignea* aerial parts extract

**Fig. 2:**
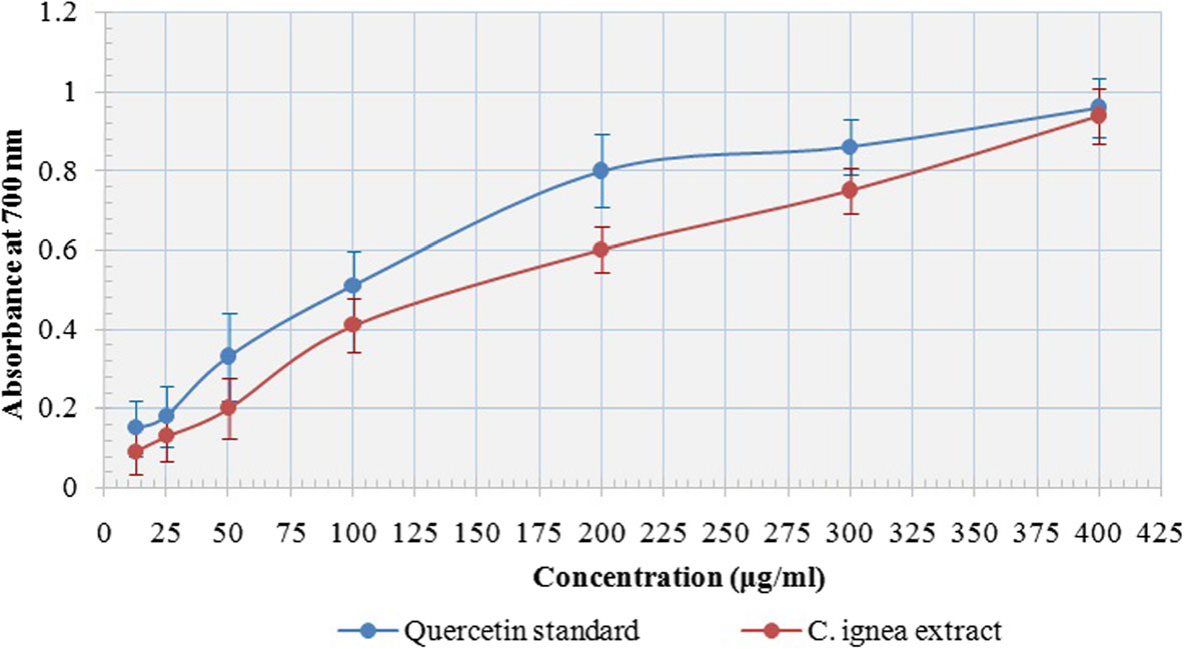
Reducing power of *C. ignea* aerial parts extract compared with quercetin as standard. Results are given as mean ± SD of three replicate analysis

The reducing power of phenolic compounds serves as good indicator of its antioxidant activity. Figure 2 showed that *C. ignea* extract had concentration-dependent reducing power. Also, it had an appreciable reducing power when compared to standard quercetin.

### Safety of *C. ignea* aerial parts extract

In this study, no mortality, no important changes in body weight and behavior (ataxia, hypoactivity, and hyperactivity) were observed in rats during the period of 72 h study up to the maximum dose (5 g/kg). 250 and 500 mg/kg doses of the extract were selected for the antiulcer studies. One week treatment with 250 or 500 mg/kg of *C. ignea* extract revealed insignificant changes in the liver and kidney functional tests (Table 2) and recorded irrelevant alterations in all parameters under the present investigation.

**Table 2:** Effect of *C. ignea* aerial parts extract on the liver and kidney function tests

Groups	ASTIU/L	ALTIU/L	ALPIU/L	Ureamg/dl	Creatininemg/dl
Control	38.75 ± 8.37	7.50 ± 3.06	59.37 ± 14.62	47.09 ± 9.08	0.58 ± 0.24
*C. ignea* 250	46.00 ± 8.10	8.75 ± 7.56	64.08 ± 17.05	46.00 ± 8.10	0.69 ± 0.17
*C. ignea* 500	38.25 ± 17.51	9.50 ± 4.62	55.02 ± 8.45	43.25 ± 13.10	0.67 ± 0.24

Data are represented by means for six rats ± SD in each group

### Effect of *C. ignea* aerial parts extract on ulcer index and preventive index

**Fig. 3:**
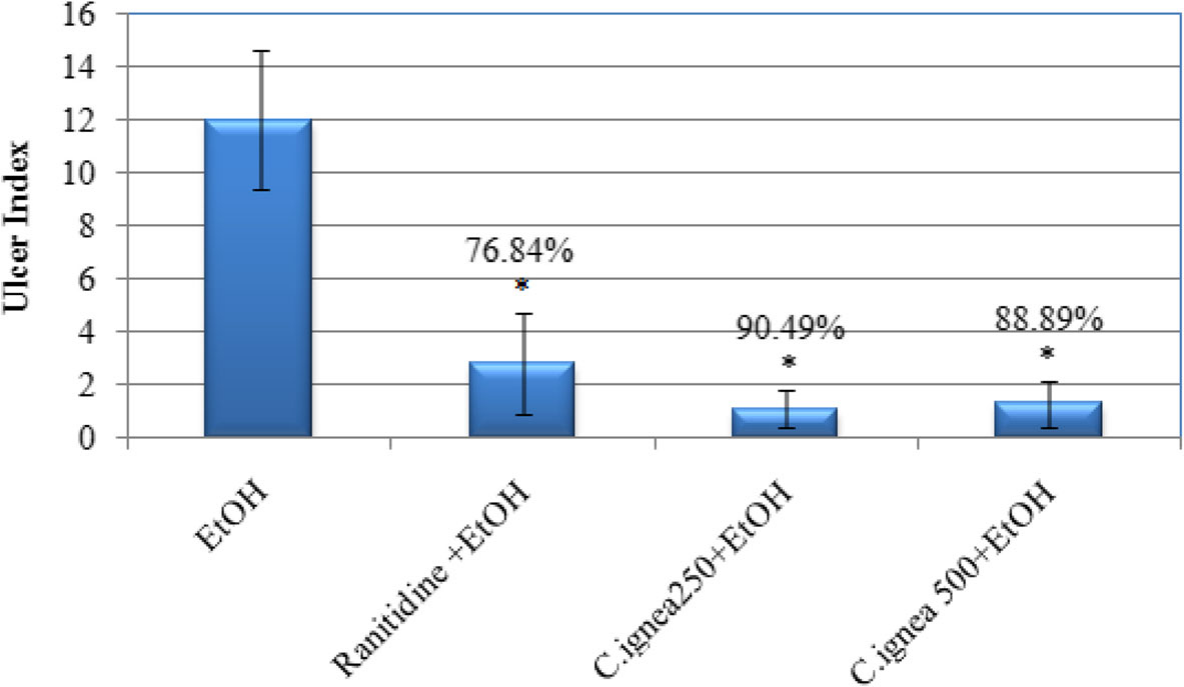
Effect of *C. ignea* aerial parts extract on ulcer index and preventive index in ethanol ulcerated rats. Each value represents the mean ± SD for six rats in each group. * *p* < 0.05 is significantly different from ethanol group

Oral administration of absolute ethanol induced gross lesions in the gastric lumen of rats with markedly high ulceration index. *C. ignea* gavaging prior to ethanol administration showed improved protection against ulceration degree. As shown in Fig. 3, both doses of *C. ignea* extract significantly reduced gastric ulcer index. The improvement in gastric ulcer index was more pronounced in *C. ignea pre*treated group than in ranitidine group. The preventive index recorded 90.49 and 88.89% for 250 and 500 mg/kg of *C. ignea*, respectively.

### Effect of *C. ignea* aerial parts extract on gastric secretion indices

Ethanol administration caused significant decrease in pH value by 34.17% with a corresponding significant increase in gastric volume of gastric content by 20-folds compared to control group. Treatments with *C. ignea* extract (250 and 500 mg/kg) produced significant increase in pH value by 17.73 and 29.91% associated with significant decrease in gastric volume by 38.78 and 27.73%, respectively as compared to ulcerated rats (Table 3). Simultaneously ethanol administration brought a significant decrease in pepsin activity of gastric juice in the ulcerated rats by 66.09% when compared with the normal control. Pepsin activity was significantly increased after treatment with 250 and 500 mg/kg of *C. ignea* extract by 19.25 and 62.12%, respectively, compared to ulcerated rats.

**Table 3:** Effect of *C. ignea* aerial parts extract on pH, volume and pepsin activity of gastric juice in various experimental groups

Groups	Gastric pH	Gastric Volume (mL)	Pepsin activity (U/mL)
Control	7.11 ± 1.99	0.12 ± 0.02	2997.20 ± 213.84
EtOH	4.68 ± 0.58^a^	2.63 ± 0.63^a^	1016.33 ± 218.03^a^
Ranitidine + EtOH	6.12 ± 1.86^ab^	1.58 ± 0.39^ab^	1382.33 ± 235.42^ab^
*C. ignea* 250	6.75 ± 0.97^b^	0.20 ± 0.14^b^	2928.83 ± 293.80^b^
*C. ignea* 250 + EtOH	5.51 ± 1.93^ab^	1.61 ± 0.34^ab^	1212.00 ± 369.84^a^
*C. ignea* 500	6.87 ± 0.83^b^	0.15 ± 0.04^b^	3148.75 ± 452.45^b^
*C. ignea* 500 + EtOH	6.08 ± 1.73^ab^	1.91 ± 0.61^ab^	1647.76 ± 311.09^ab^

Data are represented by means ± SD for six rats in each group. Statistically significant difference is expressed at *p*<0.05. ^a^ significantly different from normal control group, ^b^ significantly different from ethanol group

### Effect of *C. ignea* aerial parts extract on serum tumor necrosis factor-alpha, gastric mucosal myeloperoxidase activity and nitric oxide content

**Fig. 4:**
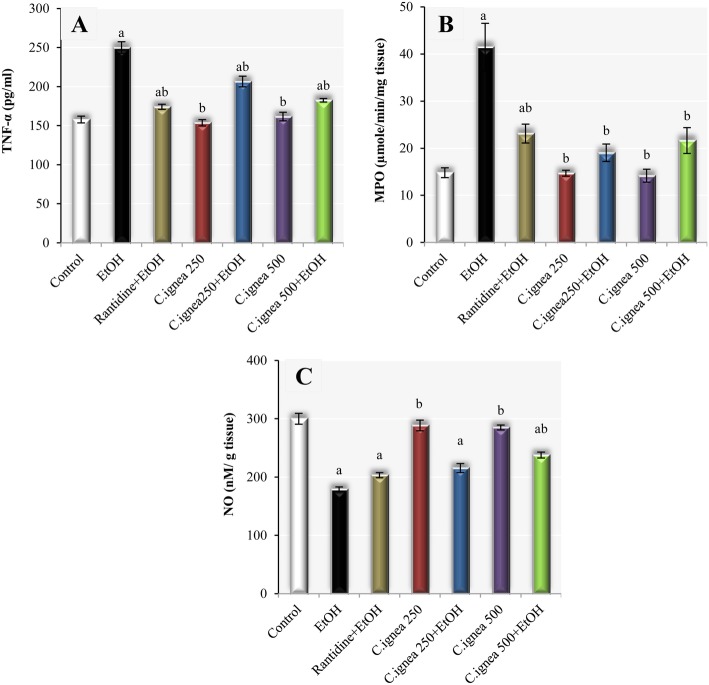
Effect of *C. ignea* aerial parts extract on (**a**): serum TNF-α, (**b**): gastric MPO and (**c**): gastric NO in various experimental groups. Each value represents the mean ± SD for six rats in each group. Statistically significant difference is expressed at *p*<0.05. ^a^ significantly different from normal control group, ^b^ significantly different from ethanol group

As shown in Fig. 4, TNF-α level and MPO activity were significantly increased in the ethanol group by 58.39 and 180.20%, respectively, conversely, NO was significantly decreased by 40.30% as compared to the normal group. On the other hand, TNF-α and MPO significantly reduced by 17.6 and 54.08%, respectively in ulcerated group pretreated with 250 mg/kg of extract in comparing with ethanol group. However, NO content was insignificantly affected. Pre-treatment with *C. ignea* at dose of 500 mg/kg showed significant improvement in TNF-α, MPO and NO by 27.20, 47.81 and 32.78% respectively. It is worth mentioning, improvement in MPO activity was more obvious in groups treated with *C. ignea* extract than in the animals treated with ranitidine.

### Effect of *C. ignea* aerial parts extract on gastric antioxidants and MDA

**Fig. 5:**
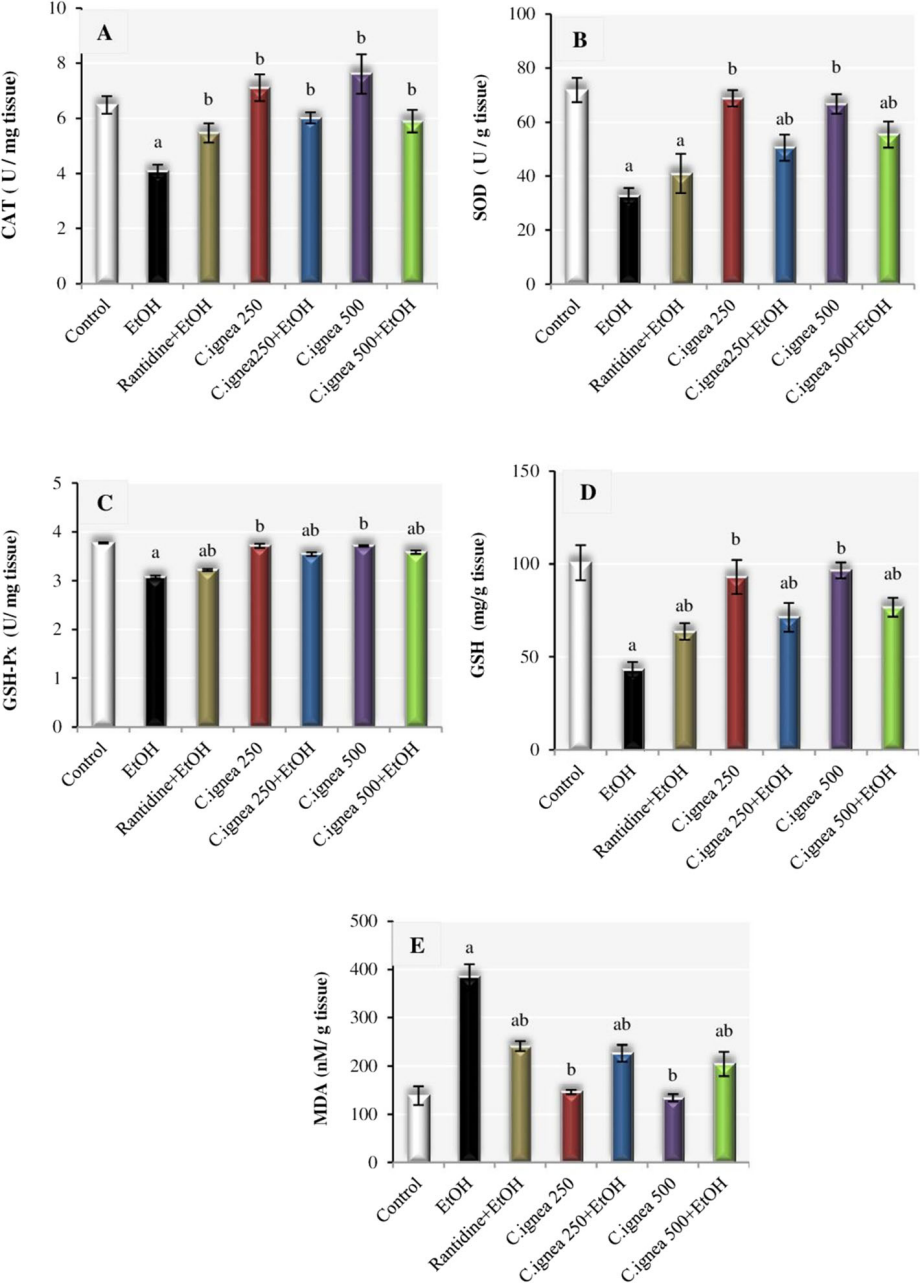
Effect of *C. ignea* aerial parts extract on (**a**): CAT, (**b**): SOD, (**c**): GSH-Px, (**d**): GSH, (**e**): MDA in gastric tissue of various experimental groups. Each value represents the mean ± SD for six rats in each group. Statistically significant difference is expressed at *p*<0.05. ^a^ significantly different from normal control group, ^b^ significantly different from ethanol group

In the present study, ulcerated rats recorded significant decrease in gastric CAT (37.13%), SOD (54.46%), GSH-Px (18.06%), GSH (57.13%), contents, and typically showed significant increase in MDA level (1.77 fold) as compared to normal group. On the other hand, pre-treatment with *C. ignea* extract at dose of 250 mg/kg significantly increased CAT, SOD, GSH-Px, GSH contents by 47.54, 54.46, 14.6, 65.24%, respectively, and significantly decreased MDA level by 41.10% as compared to ethanol group. Pre-treatment with *C. ignea* extract at dose of 500 mg/kg recorded significant improvement in all antioxidant markers with almost highest improvement percentages. It showed improvement by 69.34, 15.89, 77.75, 46.78% for SOD, GSH-Px, GSH and MDA respectively as compared to ulcerated rats. However, CAT enzyme recorded 44.60% improvement only (Fig. 5).

### Pathological findings on the gastric mucosa

#### Macroscopical findings

**Fig. 6:**
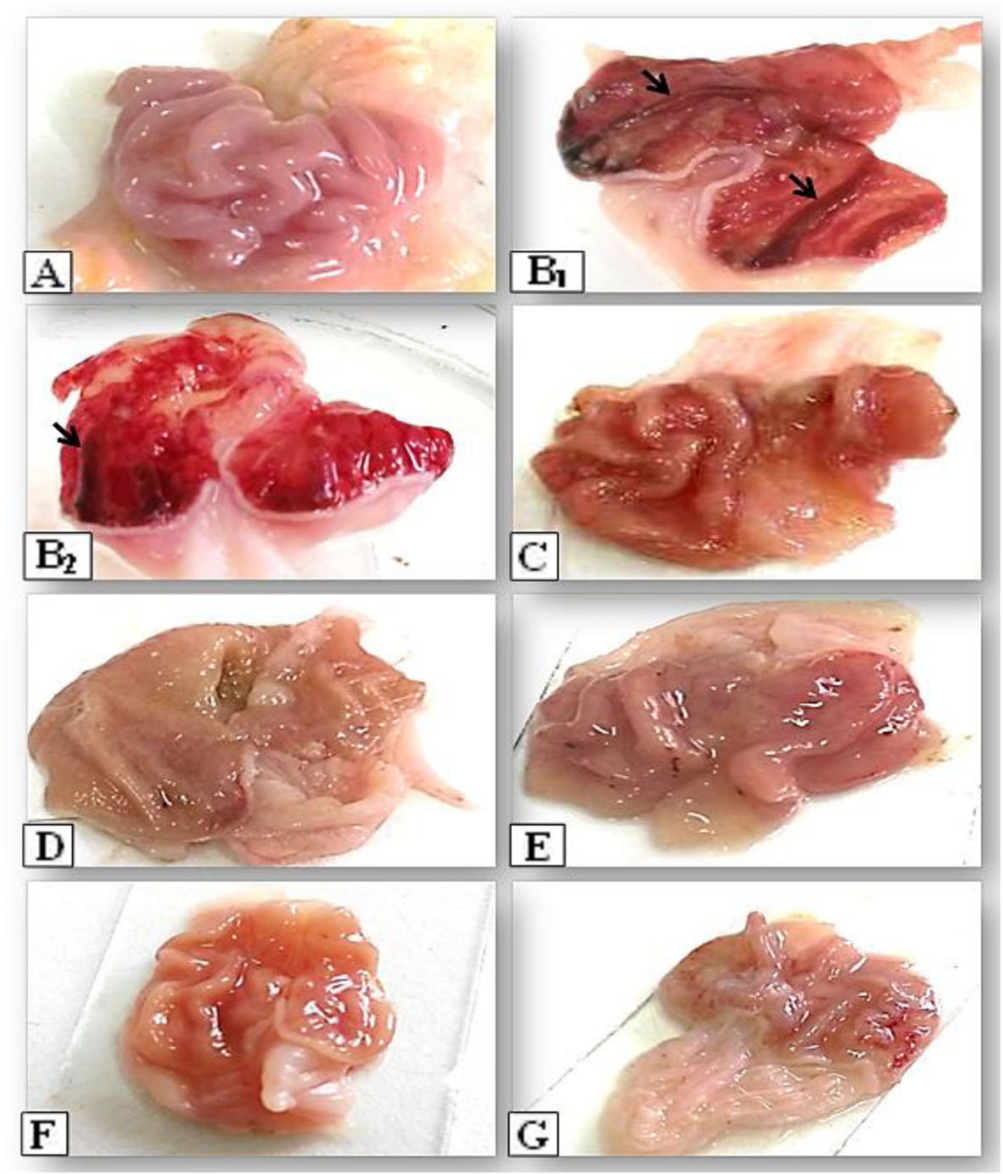
Photograph of rat stomach showing protective effect of *C. ignea* aerial parts extract on the ethanol induced gastric ulceration in rat exposed to an experimental model as; Control (**a**), EtOH (**b**), Ranitidine (**c**), *C. ignea* 250 mg/kg (**d**), EtOH + *C. ignea* 250 mg/kg (**e**), *C. ignea* 500 mg/kg (**f**), EtOH + *C. ignea* 500 mg/kg (**g**). **a** showed normal intact gastric mucosa. **b**_**1**_ and **b**_**2**_ revealed severe dark red submucosal hemorrhagic strikes (black arrows). In (**c)** congestion in gastric mucosa appeared. In (**d** and **f**) the gastric mucosa exhibited normal gross appearance with slight hyperemia and normal thickening in mucosa. In (**e** and **g**) the gastric mucosa returned back to normal gross appearance with no lesions of hemorrhages or congestion

The macroscopic examination for stomach of the control rats showed completely healthy pink color gastric mucosa with normal mucosal thickening (Fig. 6a). The ethanol treated group grossly exhibited exaggerated tissue reactions as; severe dark red submucosal hemorrhagic strikes with different sizes associated with mucosal thickening (Fig. 6b1, b2). The ranitidine treated group revealed that; the hemorrhagic vascular response became negligible but the mucosa still highly congested and swollen (Fig. 6c). Groups pretreated only with *C. ignea* extract at both doses showed no toxic or deteriorating effects on the gastric mucosa of the treated rats as; Normal non congested gastric mucosa with normal thickening were appeared (Fig. 6d, f). Rats pretreated with *C. ignea* extract at both doses before ethanol intoxication exhibited good protective level of *C. ignea* extract against ethanol effects as; mucosa color appeared normal pink with no thickening, also hemorrhages and congestion have not been noticed (Fig. 6e, g).

#### Histopathological findings

**Fig. 7:**
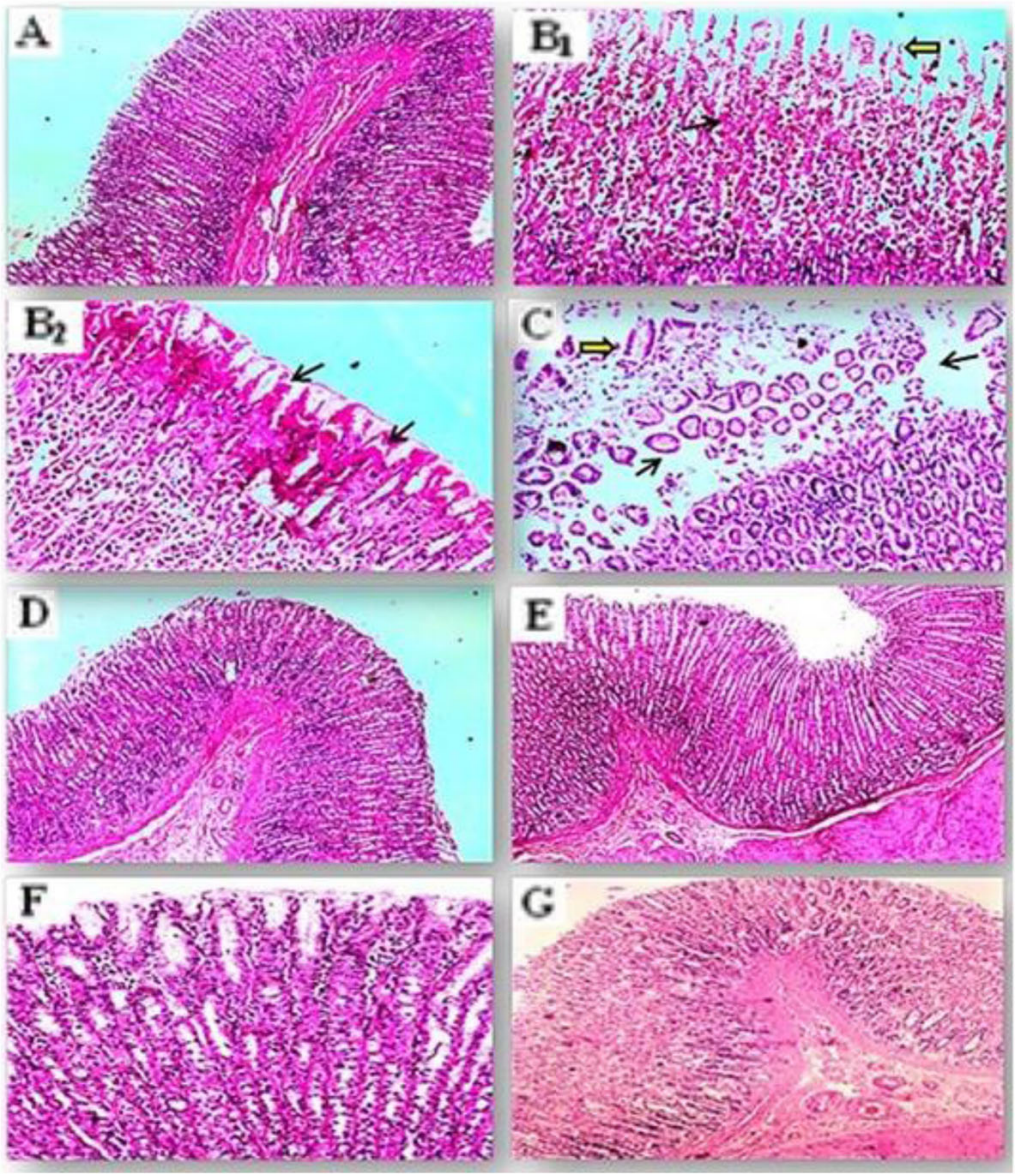
Photomicrograph from stomach tissues for the protective effects of *C. ignea* aerial parts extract against ethanol induced gastric ulceration in rats. Histopathological tissue sections were stained with H&E stain. In control rats; gastric mucosa showed intact villi with no hemorrhages or exfoliations (**a** × 100). EtOH group; showed severe intervillus hemorrhages in gastric mucosa (black arrow) accompanied with severe desquamations in mucous cells (yellow arrow) (**b**_**1**_ × 200). Severe coaggulative necrosis in gastric villi (black arrows) was noticed (**b**_**2**_ × 200). In Ranitidine group; mucous cell desquamations of gastric villi (yellow arrow) and pyloric glands (black arrows) are pronounced (**c** × 100). In *C. ignea* 250 mg/kg and 500 mg/kg groups respectively the gastric tissue is histologically normal (**d** and **f** × 100). EtOH + *C. ignea* 250 mg/kg and EtOH + *C. ignea* 500 mg/kg groups; the lining epithelium of the gastric mucosa showed no exfoliations in the mucous cell, also no submucosal hemorrhages (**e** and **g** × 100)

Control rats showed that; the villi of gastric mucosa were intact with no signs of hemorrhages or congestion; also there is no exfoliation in the mucosal epithelium (Fig. 7a). The ethanol treated group showed multifocal edema and mononuclear infiltration of inflammatory cells in the submucosal area. Moreover severe inter-villus hemorrhages associated with severe exfoliations in the mucous cells of the gastric mucosa (Fig. 7b_1_). Some other areas of gastric mucosa exhibited severe coaggulative necrosis (Fig. 7b_2_). The ranitidine treated group revealed that; the extravasation of RBCs in the core of gastric villi among the intervillus spaces became negligible. More over the desquamation of the mucous cells of the gastric mucosa and complete separation in the pyloric glands are still pronounced even with the ranitidine treatment (Fig. 7c). Groups pretreated only with *C. ignea* extract at both doses did not show any pathological changes or deteriorations on the gastric tissue (Fig. 7d, f). In ulcerated rats pretreated with *C. ignea* extract at both doses before ethanol intoxication; the gastric mucosa returned back again to normal intact mucosa with no hemorrhages or congestion. No desquamation in pyloric glands (Fig. 7e, g).

## Discussion

Alcohol consumption has been considered as a leading cause of gastric ulcer in humans; hence, researchers used the animal model of gastric injury induced by ethanol to simulate conditions that humans may be exposed, to study the antiulcer efficacy of natural products or new therapeutics intended to be used for gastric protection [4]. Oral administration of absolute ethanol in the animal model is destructive to stomach tissue, since it penetrates rapidly and easily into the gastric mucosa, producing gastric lesions [33]. Such lesions characterized by extensive submucosal edema, hemorrhage, desquamation of epithelial cells and infiltration of inflammatory cells, which are typical characteristics of alcohol injury in humans [34, 35].

The current study was designed for the first time, to study gastroprotective effect of aqueous ethanolic extract of *C. ignea* aerial parts against ethanol-induced gastric ulcer in comparison to ranitidine, which is widely approved and used for gastric ulcer treatment. This study is based on our phytochemical screening of this extract which revealed the presence of flavonoids, tannins, triterpenoids and saponin. These phytoconstituents, particularly flavonoids and tannins, were previously established to be among the possible cytoprotective agents involved in reducing gastric ulcer [36, 37].

In the present study, a high degree of ulceration was observed in rats treated with absolute ethanol. This was clearly confirmed by macroscopical and histopathological findings which revealed severe hemorrhage, appeared as severe congestion in the lamina propria submucosa and inter-villus extravasation of RBCs extended among the mucosal villi of the gastric tissue. These findings could be due to ethanol toxicity which causes decrease in the coagulopathy process which leads to the continuity of hemorrhage [38]. Hu et al. [39] reported that hemorrhagic shock induced by ethanol toxicity in lab animals is followed by alterations in the level of some pro-inflammatory and inflammatory mediators. Moreover, severe coaggulative necrosis was observed in some areas of gastric mucosa of ethanol treated rats. This result is in accordance with Liu et al. [4] and Li et al. [40], who stated that ethanol administration could induce gastric micro-vessel disturbance and blood flow stasis which finally lead to necrotic gastric injury. Pre-treatment of rats with *C. ignea* extract significantly reduced the ulcer index at both doses compared to ulcerated group. Moreover, ulcerated animals pre-treated with *C. ignea* showed a better reduction in ulcer index than the standard drug ranitidine, indicating that *C. ignea* could be valuable in healing gastric ulcer. This result is in line with the study of Abebaw et al. [41], who reported similar effect for *Osyris quadripartite* Decne extract as compared to ranitidine.

Lüllmann et al. [42] stated that elevated concentration of the hydrogen ion is an aggressive factor facilitating gastric damage via decreasing pH in gastric juice. The present study showed significant reduction in gastric pH level in ethanol treated rats comparing to normal control group. *C. ignea* pre-treatment in ethanol-ulcerated groups significantly improved gastric pH levels with simultaneous decreases in gastric secretion in comparison to ethanol group. The efficiency of *C. ignea* extract in increasing gastric pH could be attributed to the presence of flavonoids in the extract. According to Zhao et al. [43] and Liu et al. [44], flavonoids have a main role in the mechanism of gastro-protection by rising pH of gastric juice. Moreover, our results showed that pre-treatment with *C. ignea* extract had similar effects on gastric pH as the reference ranitidine drug, which has a great ability to decrease stomach acid production and neutralize stomach acidic environment. Furthermore, our study showed that, ethanol ulcerated rats have significant reduction in pepsin activity in comparing to normal group and this is in agreement with Puurunen [45] who clarified that, high concentrations of ethanol can reduce peptic activity due to its ability to inhibit pepsinogen activation to pepsin. On the other hand, *C. ignea* pre-treatment improved pepsin activity in gastric secretion in dose dependent manner, indicating that *C. ignea* extract has the ability to regulate ethanol effect on peptic activity.

Inflammatory response is one of the characteristics of gastric ulcer which promotes gastric mucosal injury through the migration of macrophages and leukocytes into the ulcerated and the surrounding areas [46]. TNF-α is a major pro-inflammatory cytokine released from migrated macrophages during inflammation [47]. It stimulates neutrophil infiltration in gastric inflamed areas [48] and suppresses the gastric microcirculation around ulcerated mucosa and delays gastric ulcer healing [49]. The present data indicated that ethanol administration induced inflammatory response as evidenced by the marked increase in serum level of TNF-α as compared to control group. This result is consistent with previous reports of Li et al. [40] and El-Hussieny et al. [50] who reported an increase in gastric tissue pro-inflammatory cytokines due to ethanol administration. On the other hand, a dose-dependent reduction in TNF-α level was observed in the ulcerated groups pretreated with *C. ignea*, and this may be attributed to its anti-inflammatory effect. This result was confirmed by our histopathological findings which revealed decreased inflammatory responses by *C. ignea* pre-treatment.

Increased neutrophils infiltration into the gastric mucosa due to ethanol administration is assessed by elevation of the gastric MPO activity released from neutrophils [51, 52]. This was observed in the present work by marked increase in MPO activity in the stomach of rats treated with ethanol. Inhibition of neutrophil infiltration into ulcerated gastric tissues is a vital anti-inflammatory mechanism by which anti-ulcer agents can improve the healing process of gastric ulcer and protect against it [53]. Pre-treatment with *C. ignea* extract in ulcerated rats caused a significant and dose dependent reduction in neutrophil infiltration into the gastric mucosa as evidenced by suppression of MPO activity, demonstrating its anti-ulcer effect.

Nitric oxide, derived from constitutive nitric oxide synthase, is a vital endogenous mediator of mucosal defense and plays a significant role in the maintenance of normal gastric mucosal integrity [54]. This role of NO may be due to its ability to reduce neutrophil infiltration [55] and to influence blood flow in gastric tissues during the healing process of gastric ulcer [56]. In the present study, ethanol ulcerated group showed significant reduction in gastric NO levels in comparing to control group. This finding is in accordance with Goswami et al. [57] and Nordin et al. [58]. On the other hand, ulcerated rats pre-treated with *C. ignea* displayed marked increase in NO level, indicating its anti-ulcer efficacy. According to Abdulla et al. [59] keeping normal levels of nitric oxide is one of the main mechanisms used to protect gastric mucosa against harmful effects of ethanol.

Laine et al. [60] stated that reactive oxygen species (ROS) generated by neutrophils in gastric mucosa has a critical role in the gastric mucosal injury. Later, Al Rashdi et al. [61] and Kan et al. [62] reported that elevated production of ROS and depletion of antioxidants are involved in the pathophysiology and development of ethanol-induced gastric ulcer. According to Yu et al. [63], accumulation of ROS leads to lipid peroxidation as a result of their reaction against cell membrane. Our data revealed that, ethanol administration significantly reduced the activity levels of antioxidant enzymes (CAT, SOD and GSH-Px) and increased the concentration of MDA with concomitant depletion in GSH concentration in the gastric tissue of ethanol group, this is in the same line with the previous studies of Sidahmed et al. [64]. On the other hand, pre-treatment of *C. ignea* extract in ulcerated groups has a great efficacy in preventing free radical mediated oxidative damage by enhancing the activity of antioxidant enzymes (CAT, SOD and GSH-Px) and restoring the depleted GSH levels together with reducing MDA levels. This antioxidant effect of the *C. ignea* extract could be attributed to its strong free radical scavenging activity due to the presence of a significant amount of, the powerful antioxidants, flavonoids and phenolic compounds. This is consistent with Mei et al. [53] who established that one of the mechanisms responsible for the healing of ulcer is scavenging of ROS. Our study showed that *C. ignea* extract had strong antioxidant effect, which is comparable to that of ranitidine. Ahmadi et al. [65] previously reported that therapeutic effect of ranitidine on ulcer could be related to its antioxidant capacity through oxidative stress reduction mediated by scavenging of hydroxyl radical.

## Conclusions

The results of the present study demonstrated that the aqueous ethanolic extract of *C. ignea* aerial parts at both doses attenuated ethanol-induced gastric ulcer through its antioxidant and anti-inflammatory effects. This gastroprotective efficiency of *C. ignea* aerial parts extract could be possibly attributed to the presence of wealthy phytoconstituents as total polyphenols, flavonoids and tannins. Therefore, *C. ignea* could be used as a promising anti-ulcer agent in the treatment of gastric ulcers due to its comparable anti-ulcer effect to that of ranitidine. However, further researches should be taken to further explore the underlying mechanisms of action.

## Data Availability

All the data generated in this current work are included in the ‘Result and Discussion’. Raw data supporting the findings of the current work are available from the corresponding author on reasonable request.

## Abbreviations

ALP: Alkaline phosphatase
ALT: Alanine transaminase
AST: Serum aspartate transaminase
CAT: Catalase
GSH: Reduced glutathione
GSH-Px: Glutathione peroxidase
H&E: Hematoxylin and eosin
MDA: Malondialdehyde
MPO: Myeloperoxidase
NO: Nitric oxide
ROS: Reactive oxygen species
SOD: Superoxide dismutase
TNF-α: Tumor necrosis factor-alpha
